# Oestrogen receptor beta isoform expression in sporadic colorectal cancer, familial adenomatous polyposis and progressive stages of colorectal cancer

**DOI:** 10.1186/s12885-017-3688-4

**Published:** 2017-11-13

**Authors:** Paulo Roberto Stevanato Filho, Samuel Aguiar Júnior, Maria Dirlei Begnami, Hellen Kuasne, Ranyell Matheus Spencer, Wilson Toshihiko Nakagawa, Tiago Santoro Bezerra, Bruna Catin Kupper, Renata Maymi Takahashi, Mateus Barros Filho, Silvia Regina Rogatto, Ademar Lopes

**Affiliations:** 1 Colorectal Tumor Nucleus of the Pelvic Surgery Department, A.C. Camargo Cancer Center, São Paulo, SP Brazil; 2 Department of Pathology, A.C. Camargo Cancer Center, São Paulo, SP Brazil; 3 CIPE - International Center for Research, A. C. Camargo Cancer Center, São Paulo, Brazil; 4 Department of Clinical Genetics, Vejle Sygehus, Vejle and Institute of Regional Health Research, University of Southern Denmark, Odense, Denmark; 5 Colorectal Tumor Nucleus of the A.C. Camargo Cancer Center, R. Professor Antônio Prudente, 211 Liberdade, São Paulo, São Paulo, SP CEP 01509–010 Brazil

**Keywords:** Colorectal cancer, ERβ isoforms, Oestrogen receptor, Familial adenomatous polyposis, Hormone receptors

## Abstract

**Background:**

Among the sex hormones, oestrogen may play a role in colorectal cancer, particularly in conjunction with oestrogen receptor-β (ERβ). The expression of ERβ isoform variants and their correlations with familial adenomatous polyposis (FAP) syndrome and sporadic colorectal carcinomas are poorly described.

**Methods:**

This study aimed to investigate the expression levels of the ERβ1, ERβ2, ERβ4 and ERβ5 isoform variants using quantitative RT-PCR (921 analyses) in FAP, normal mucosa, adenomatous polyps and sporadic colorectal carcinomas.

**Results:**

Decreased expression of ERβ isoforms was identified in sporadic polyps and in sporadic colorectal cancer as well as in polyps from FAP syndrome patients compared with normal tissues (*p* < 0.001). In FAP patients, ERβ1 and ERβ5 isoforms showed significant down-expression in polyps (*p* < 0.001) compared with matched normal tissues. However, no differences were observed when sporadic colorectal carcinomas were compared to normal mucosa tissues. These findings suggest an association of the ERβ isoform variants in individuals affected by germline mutations of the APC gene. Progressively decreased expression of ERβ was found in polyps at early stages of low-grade dysplasia, followed by T1-T2 and T3-T4 tumours (*p* < 0.05). In sporadic colorectal cancer, the loss of expression was an independent predictor of recurrence, and ERβ1 and ERβ5 expression levels were associated with better disease-free survival (*p* = 0.002).

**Conclusion:**

These findings may provide a better understanding of oestrogens and their potential preventive and therapeutic effects on sporadic colorectal cancer and cancers associated with FAP syndrome.

**Electronic supplementary material:**

The online version of this article (10.1186/s12885-017-3688-4) contains supplementary material, which is available to authorized users.

## Background

Colorectal cancer (CRC), the second and third most commonly diagnosed cancer in women and men, respectively, presents a variable geographical incidence that has been attributed to differences in diet and environmental exposure [1]. These variations occur in conjunction with a genetically determined background of susceptibility. The incidence of CRC is substantially higher in men and women greater than 50 years of age, although the basis for this difference is unknown. Sex steroids have been considered a contributing factor because high parity, early age at first pregnancy, oral contraceptive use and oestrogen replacement therapy are associated with decreased risk of CRC [2–5]. In addition, younger women (<45 years of age) with CRC have better overall survival than men of the same age [6].

Familial adenomatous polyposis syndrome (FAP), a precancerous colorectal condition, is an inherited disease caused by a germline mutation in the adenomatous polyposis coli (*APC*) gene [7]. In FAP, many neoplastic lesions of different stages are typically found within the same individual, thus providing a better understanding of the adenoma-carcinoma progression. The fact that these polyps appear in the second decade of life (i.e., during puberty) and show increased size and number during adolescence (i.e., at the peak of production) also suggests that sex steroids may act as a cofactor in the development of colorectal polyps and in carcinogenesis linked to FAP [8].

Evidence from several studies suggests that among the sex hormones, oestrogen may play a role in colorectal cancer, particularly through oestrogen receptor-β (ERβ) [9–13]. However, since the discovery of this receptor in 1996 [14], research efforts have been focused on defining its biological function, which remains poorly understood. Several alternative splicing isoforms of the oestrogen receptor beta gene (*ESR2)* occur (transcript variants a, b, d, f, g, h, i, k, l; ERβ isoforms 1–5), and these isoforms have been detected in both normal and malignant cells [15–18]. The expression of *ESR2* splicing variants in colon cancer cells was first reported in 2001 [19]. To our knowledge, this is the first study in which the expression levels of *ESR2* variants in FAP, adenomatous polyps and sporadic colorectal carcinomas have been evaluated and compared with normal mucosa, tumour stage and prognosis data. The goal of this study was to provide a better understanding of oestrogens and their potential effects on sporadic and hereditary colorectal tumours.

## Methods

### Patients

Fresh frozen tissue samples from 98 patients with sporadic CRC were retrospectively collected in the Institutional biobank between the years 2005 and 2016; 48 of the patients had stage T1/T2 tumours, and 50 had stage T3/T4 tumours. As a reference, paired histologically normal mucosa was obtained from 52 cases. Formalin-fixed, paraffin-embedded (FFPE) tissue samples were obtained from 52 sporadic polyps. In addition, 64 FFPE polyps and 41 FFPE adjacent normal tissues were obtained from 41 FAP patients, between the years 2000 and 2016. All specimens were submitted to macrodissection and histology confirmation. Tumor samples were composed of at least 70% of epithelial cells, and normal tissues presented more than 90% of epithelial cells. In total, 307 samples were evaluated, with 921 analyses of three transcripts and their associated isoform pairs.

Patients undergoing neoadjuvant treatment and those with other forms of hereditary CRC or inflammatory bowel disease were excluded from the study. The Institutional Review Board of the A.C. Camargo Cancer Center, Sao Paulo, Brazil, approved this study (CEP01453/10).

The histopathological classification of the tumours and the clinical staging followed the recommendations of the WHO International Classification of Diseases for Oncology [20] and the Tumour-Node-Metastasis staging system (TNM) [21], respectively. The medical records of the patients were examined to obtain detailed clinical and pathological data. FAP was diagnosed in individuals with more than 100 adenomatous colon polyps or with *APC* mutations.

The routine follow-up of patients with sporadic colorectal tumours treated at the institution included quarterly clinic visits during the first two years, with laboratory tests, assessment of tumour markers, chest X-ray and ultrasound or abdominal sectional imaging (tomography) performed at alternating visits. These procedures were performed every six months from the third to the fifth year and annually thereafter. Colonoscopy was performed at the first, third and fifth years postoperatively. In the event of recurrence, tests were requested for re-staging and treatment planning. Positron emission tomography (PET-CT) was performed in cases considered to have higher risk of metastasis.

The mean follow-up time was 70.1 months, and the median was 58.6 months (1.67 to 188.7 months). The FAP patients were carriers of the classic phenotype (>100 adenomatous polyps) and 53.6% were male with a median age at syndrome diagnosis of 31 years (19 to 56 year old). The polyps were located in the colon, and histology analysis showed low-grade dysplasia. Demographic, clinical and pathological characteristics and treatment of individuals with sporadic cancer (*N* = 98) are summarised in Table 1.

**Table 1:** Sample distribution according to the demographic and clinical variables of individuals with cancer

Variable	Category	Frequency (%) *N* = 98
Gender	Male	53 (54.1)
	Female	45 (45.9)
Age (years)	Variation	41–88
	Median	58.6
	Mean (Standard Deviation)	65.45 (10.2)
Age range (years)	≤65	52 (53.1)
	>65	46 (46.9)
Location	Proximal	30 (30.6)
	Distal	68 (69.4)
Topography	Colon	82 (83.7)
	Upper rectal	16 (16.3)
Follow-up time (months)	Variation (months)	1.7–188.7
	Median	58.6
	Mean (Standard Deviation)	70.15 (42.3)
Status	Living without disease	78 (79.5)
	Living with disease	2 (2.04)
	Death from other causes	3 (3.06)
	Death from disease	15 (15.3)
Recurrence	No recurrence	77 (78.5)
	Local	1 (1.02)
	Liver	13 (13.2)
	Lung	4 (4.08)
	Peritoneum	3 (3.0)
T Stage	T1/T2	48 (48.9)
	T3/T4	50 (51.1)
N Stage	N0	58 (59.2)
	N+	40 (40.8)
Overall TNM stage	S I	39 (39.8)
	S II	18 (18.4)
	S III	27 (27.6)
	S IV	14 (14.3)
Histological grade	Well differentiated (G1)	08 (8.2)
	Moderately differentiated (G2)	84 (85.7)
	Poorly differentiated (G3)	06 (6.3)
Blood embolization	No	95 (96.9)
	Yes	3 (3.1)
Perineural invasion	No	89 (90.8)
	Yes	9 (9.20)
Lymphatic embolization	No	85 (86.7)
	Yes	13 (13.3)
Adjuvant chemotherapy	No	52 (53.7)
	Yes	46 (46.7)
Adjuvant CT scheme	No chemotherapy treatment	52 (53.7)
	FOLFOX	28 (28.5)
	5 FU + LV	07 (7.14)
	FOLFOX/FOLFIRI + Bevacizumab	05 (5.10)
	FOLFIRI + Cetuximab	1 (1.02)
	XELODA/XELOX	5 (5.10)

*CT* chemotherapy, *5-FU + LV* 5-Fluorouracil + Leucovorin (folinic acid), *FOLFOX* Leucovorin + Oxaliplatin + Oxaliplatin, *FOLFIRI* Fluorouracil + Leucovorin + Irinotecan, *XELODA/XELOX* Capecitabine combined with Oxaliplatin

### Methods

#### RNA extraction

Total RNA was extracted from macrodissected fresh frozen tissues and FFPE tissue samples using a QIAsymphony RNA Kit (Qiagen) and a RecoverAll Total Nucleic Acid Isolation Kit (Ambion) according to the manufacturer instructions. The RNA quantity and quality from tissue samples (FFPE and fresh frozen) were evaluated using a NanoDrop ND-1000 Spectrophotometer (v.3.0.1, Labtrade). The RNA quality from fresh frozen tissue samples was also evaluated using the Bioanalyser Agilent RNA 6000 Nano LabChip kit (Agilent 2100 bioanalyser) (Additional file 1: Table S1).

#### Selection of the reference genes

The Cancer Genome Atlas (TCGA: level 3 colon tumour RNA sequencing database) was used to select the reference genes for RT-qPCR normalisation. The most stable genes were identified among the 30 potential reference transcripts (Applied Biosystems TLDA test) using 41 surrounding normal tissues and 285 colon adenocarcinomas. A standard deviation (SD) ranking was conducted for each gene in all samples, and the *p*-value (unpaired t-test for unequal variances) was calculated comparing the normal and tumour samples. The lowest SDs and highest *p*-values were ranked; the *PUM1*, *POP4* and *EIF2B1* genes had the highest rankings (lowest variation between samples and without differences between the normal and tumour groups). The expression levels of *ESR2* were evaluated by RT-qPCR using the *PUM1*, *POP4* and *EIF2B1* genes as a reference.

#### Reverse transcription quantitative polymerase chain reaction (RT-qPCR)

**Fig. 1:**
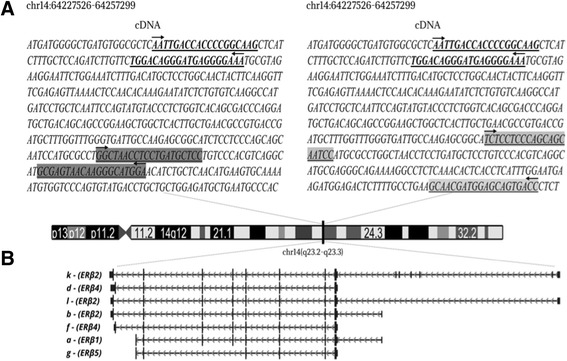
Schematic representation of the transcript variants and isoforms of *ESR2* gene evaluated in this study. **a** Three primer pairs were used to amplify the ESR2 variants. Primer pair 1, in bold, amplifies five *ESR2* transcript variants (a, b, d, f, k and l), representing the isoforms ERβ1, ERβ2 and ERβ4. Primer pair 2, highlighted in dark grey, amplifies the transcript variants a and g (ERβ1 and ERβ5). Primer pair 3, highlighted in light grey, amplifies the variants b, l and k (ERβ2). **b** The *ESR2* transcript variants are indicated by letters, followed by the correspondent isoforms. Figure modified from UCSC Genome Browser (https://genome.ucsc.edu). The nomenclature was defined according to NCBI (https://www.ncbi.nlm.nih.gov/nuccore)

Three pair of primers flanking the *ESR1* gene were designed. Primer pair 1 amplifies the transcript variants a, b, d, f, k and l (*ERβ1, ERβ2* and *ERβ4* isoforms*);* primer pair 2 amplifies transcripts a and g (*ERβ1* and *ERβ5* isoforms), and primer pair 3 amplifies the transcript variants b, l and k, which are translated into the *ERβ2* isoform (Table 2 and Fig. 1)*.* Three endogenous references (*PUM1*, *EIF2B1*, and *POP4*) were used in the RT-qPCR assays (Table 1). The primers were designed using Primer-Blast (available at http://www.ncbi.nlm.nih.gov/tools/primer-blast/), and Primer 3 was selected based on the study by Yang et al., 2016 [22].

**Table 2:** Sequences and properties of the primers used in the study

Gene	Primer 5′ - 3′	Amplicon length (bp)	Transcript variants	Encoded Isoforms
*ESR2*	F:AATTGACCACCCCGGCAAG	64	a, b, d, f, k and l	*ERβ1, ERβ2 and ERβ4*
*Primers 1*	R: TTTCCCCTCATCCCTGTCCA			
*ESR2*	F:GGCTAACCTCCTGATGCTCC	57	a and g	*ERβ1* and *ERβ5*
*Primers 2*	R: TCCATGCCCTTGTTACTCGC			
*ESR2* ^a^	F:TCTCCTCCCAGCAGCAATCC	154	b, l and k	*ERβ 2*
*Primers 3*	R:GGTCACTGCTCCATCGTTGC			
*PUM1*	F:CACAGACACCACCTCCTTCC	73		
	R:CCATTCGTGAGTCCTCCCAG			
*EIF2B1*	F:ACCTGTCTTCATCCTCCCCT	71		
	R:GCTGCTTTTCGCCTGCATC			
*POP4*	F:TTACCTGCTTTCCCGCTGAG	114		
	R:GGCTAGGAAGCTACAGCACC			

bp: base pairs ^a^This primer sequence was obtained from ref. [22] F: forward; R: reverse

Total RNA samples were digested with 1 U of DNase I (amplification grade, Life Technologies) in 10X DNase I Reaction Buffer. The reactions were performed in a PTC-100 thermal cycler (Peltier-Effect Cycling - MJ Research) for 15 min at room temperature; the enzyme was then inactivated by heating at 70 °C for 10 min. The cDNA synthesis was performed in a final volume of 20 μL containing 5X first-strand buffer (250 mM Tris–HCl pH 8.3, 375 mM KCl and 15 mM MgCl_2_), 10 mM of each dNTP, 0.5 μg/μL oligo (dT), 0.1 M dithiothreitol and 200 U of reverse transcriptase (SuperScriptTM III, Invitrogen). Reverse transcription was performed at 42 °C for 60 min followed by inactivation at 70 °C for 15 min. The ABI Prism 7900 Sequence Detection System automatic thermocycler was used for RT-qPCR amplification. All reactions were assembled by robotic pipetting into 384-well plates with QIAgility (Qiagen, Courtaboeuf, France) in a total volume of 12.5 μL of Power SYBR Green Master Mix (Applied Biosystems, Foster City, CA, USA), 20 ng of cDNA and 200 nM of each primer. All samples were analysed in duplicate and subjected to the following cycling conditions: an initial temperature of 95 °C for 10 min and 45 cycles of 95 °C for 15 s and 60 °C for 1 min. The amplification quality was verified by analysing the dissociation curve (specificity) to discriminate primer-dimers from low levels of transcript expression. A duplicate of no template control (NTC) was included in each PCR amplification for each primer pair, all showing negative signals (Cq = 45 or small dimer amplification detection Cq > 42). The values obtained ​​for all samples were normalised by determining the ratio of the gene of interest to the reference gene. The mean Cq (quantification cycle) was used for normalisation. Samples with a mean Cq greater than the mean + 1 SD of the geometric mean of the Cqs of the three reference genes were excluded (8 fresh frozen and 30 FFPE samples). The model proposed by Pfaffl [23] was used for data normalisation.

#### ESR2 expression in the cancer genome atlas (TCGA) database of colon cancer

*ESR2* expression data in colon carcinomas versus normal tissues (log2 + 1 reads per million) was assessed from The Cancer Genome Atlas (TCGA: level 3 RNA sequencing database) (t test). An isoform specific analysis of *ESR2* was implemented using Isoform Expression View (normal-tumor comparison) of the ISOexpresso tool (http://wiki.tgilab.org/ISOexpresso/) [22].

#### Statistical analysis

The findings obtained from five groups of tissues were compared according to the tissue type: fresh frozen or FFPE. The variants expression levels of fresh frozen sporadic colorectal tumours (ST: 98 samples) were compared with normal mucosa (NS: 52 samples). Similarly, the expression levels of FFPE samples including sporadic polyps (SP: 52 samples), normal mucosa from FAP patients (NFAP: 41 samples) and adenomatous polyps from FAP patients (PFAP: 64 samples) were compared. A comparison with all groups (NS, ST, SP, NFAP and PFAP) was also performed, independent of the tumour type (fresh frozen and FFPE) (Additional file).

Descriptive statistics were used to characterise the sample. A frequency distribution was used to describe categorical variables and measures of central tendency and variability for numerical or continuous variables. The chi-squared frequency test was used to compare categorical variables, and Fisher’s exact test was used to verify associations. Student’s t test (paired and unpaired) was used to compare continuous variables. Analysis of variance (ANOVA) was used to compare continuous variables of multiple groups. The Kaplan-Meier method was adopted to estimate the overall and disease-free survival probabilities. The difference between survival curves of the same variable was evaluated using the log-rank test. The cut-off for relative gene expression was determined using the log-rank test (maximally selected rank statistics in R) [24]. Multivariate analysis was used to predict the combined effect of independent variables on survival using the Cox model with proportional risks. The data were statistically analysed using Statistical Package for Social Sciences (SPSS) version 20.0 and GraphPad Prism 5.0 (GraphPad Software Inc., La Jolla, CA). The significance level for all statistical tests was 5%.

## Results

**Fig. 2:**
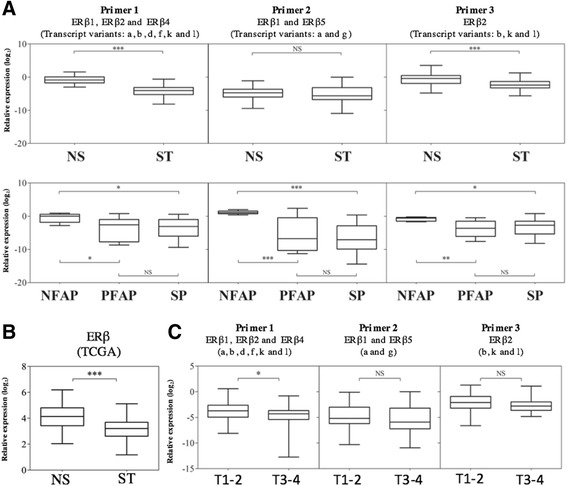
**a** *ESR2* expression levels in sporadic CRC, sporadic polyps and FAP polyps in comparison to normal tissues according to the type of tissue (fresh frozen tissues: superior graphics or FFPE: inferior graphics). **b** Comparison of ESR2 expression levels in normal and sporadic colorectal cancer of TCGA database (log2 + 1 from RNA sequencing read per million values). **c** The samples were grouped into two T stages using the TNM classification (T1/T2 vs T3/T4) with relative expression of the *ESR2* isoforms*.* NS = Sporadic normal mucosa; SP = Sporadic polyps; ST = Sporadic tumour; NFAP = Normal mucosa FAP; PFAP = Polyp FAP; TCGA = The Cancer Genome Atlas level 3) *: *p* < 0.05; **: *p* < 0.01; ***: *p* < 0.001; (Tukey’s Multiple Comparison Test and Student’s t-test)

A significant decrease of *ESR2* expression levels was observed in polyps and tumours compared to normal mucosa (fresh frozen tissues) or normal tissue from FAP patients (FFPE samples), regardless of heredity (Fig. 2A). However, differences in expression levels were found according to the group tested and the isoforms.

### Primer pair 1: Transcript variants *a, b, d, f, k,* and *l: ERβ1, ERβ2* and *ERβ4* isoforms

The levels of transcript variants a, b, d, f, k, and l, corresponding to the *ERβ1, ERβ2* and *ERβ4* isoforms, were significantly lower in sporadic tumours (ST) (*p* < 0.001; Mean Diff 3.285; 95% CI 2.780 to 3.791) compared with normal sporadic mucosa (NS) (fresh tissue) (Fig. 2a).

In FAP syndrome patients, lower expression levels of ERβ (a) mRNA were found in polyps compared with paired FAP mucosa samples (*p* < 0.05; Mean Diff 3.338; q = 3.854; 95% CI 0.3677 to 6.308). Sporadic polyps (SP) were also down-expressed when compared to FAP mucosa samples *p* < 0.05; Mean Diff 3.1; q = 3.883; 95% CI: 0.3621 to 5.839). No difference was observed in sporadic polyps (SP) versus PFAP (Fig. 2a).

Comparing all FFPE and fresh frozen tissue samples, no significant differences in mRNA expression levels were found in sporadic normal mucosa and normal FAP mucosa. However, invasive polyps and tumours showed lower *ESR2* expression levels compared with normal mucosa from both, sporadic tumours and FAP patients (Additonal file 2: Figure S1).

### Primer pair 2: Transcript variants *a* and *g: ERβ1* and *ERβ5* isoforms

No significant differences were found in the expression levels of mRNA of *ERβ1* and *ERβ5* isoforms compared with normal mucosa (NS) and tumour tissues (ST) from fresh frozen samples (Fig. 2a). However, in FFPE tissues, the normal mucosa from FAP syndrome patients showed high expression levels of *ERβ1* and *ERβ5* isoforms compared with the sporadic polyps (*p* < 0.001; Mean Diff 7.568; q = 7.381; 95% CI 4.054 to 11.08) and FAP polyps (*p* < 0.001; Mean Diff 6.4; q = 5.721; 95% CI 2.566 to 10.23) (Fig. 2a).

The analysis of all groups together (Additional file 2: Figure S1) revealed downexpression levels *ERβ1* and *β5* isoforms in sporadic tumours compared to normal mucosa of FAP patients (*p* < 0.001; Mean Diff −5.926; q = 8.04; 95% CI −8.81 to −3.04). Furthermore, the normal mucosa of FAP syndrome patients showed a higher concentration of *ERβ1* and *ERβ5* isoforms than the other tissues analysed.

### Primer pair 3: Transcript variants *b, i* and *k*: *ERβ2* isoform

*ERβ2* expression levels were significantly lower in tumour tissues (*p* < 0.001; Mean Diff 1.718; 95% CI 1.161 to 2.275) than in sporadic normal mucosa (Fresh frozen tissue). Similarly, for FFPE tissues, lower *ERβ2* mRNA expression levels were found in FAP polyps (*p* < 0.05; Mean Diff 2.944; q = 4.393; 95% CI 0.6458 to 5.243) and sporadic polyps (*p* < 0.05; Mean Diff 2.438; q = 3.945; 95% CI 0.3186 to 4.557) compared with FAP mucosa.

Considering all groups together (Additional file 2: Figure S1), sporadic normal mucosa and normal FAP mucosa showed no significant differences in *ERβ2* mRNA levels. No significant differences in *ERβ2* expression between sporadic polyps and tumour tissues were detected.

### *ERβ* expression according to the cancer genome atlas (TCGA)

RNA sequencing data from 41 normal mucosa and 285 colon adenocarcinomas were extracted from the TCGA database. A significant decrease in ERβ expression level was found in sporadic tumours (*p* < 0.001; Mean Diff 0.984 ± 0.146; 95% CI 0.697 to 1.271) (Fig. 2b). The isoform specific analysis (http://wiki.tgilab.org/ISOexpresso/) highlighted low expression levels of the variants d (*ERβ4*) and b (*ERβ2*) in tumour samples (Additional file 3: Figure S2).

### *ERβ* expression according to T stage (TNM)

T3/T4 sporadic tumours presented significantly decreased expression levels of *ERβ* when compared with T1/T2 tumours (*p* = 0.02; Mean Diff 0.87; 95% CI 0.12 to 1.63; Student’s t test). Decreased expression levels were observed only when Primer 1 was used, which amplifies a greater number of transcription variants (the a, b, d, f, k, and l variants, which produce the isoforms *ERβ1, ERβ2* and *ERβ4*) (Fig. 2c). The comparison of T stage (TNM) (T1 vs T2 vs T3 vs T4) in the four classes revealed no significant differences (one-way ANOVA and Tukey’s post hoc test).

### Disease-free survival and overall survival analysis

The time to occurrence of relapse ranged from 4.7 to 36.4 months (median 15.4 months and mean 15.6 months). The probability of recurrence-free survival was 77.4% for both 5- and 10-year survival. By univariate analysis, disease-free survival was greater in subjects ≥65 years of age and in those with normal serum carcinoembryonic antigen levels, T1-T2 tumours, absence of blood embolization, absence of lymph node or metastatic invasion, well differentiated tumour histological grade or Primer 2 (*ERβ1* and *ERβ5*) expression levels ≤ −4.5. (Table 3, Additional file 4: Figure S3). The multivariate analysis revealed that T and N stage and ERβ expression levels ≤ −4.5 are independent predictors of disease-free survival (Table 4).

**Table 3:** Probability of overall survival and disease-free survival at 5 and 10 years according to the demographic, clinical and pathological variables of sporadic colorectal cancer patients

Variable	Overall survival (%)		*p*	Disease-free survival (%)		*p*
	5-y	10-y	–	5-y	10-y	
Survival	83.9	77.1	–	77.4	77.4	–
Gender						
Male	88.3	79.5	0.220	77.6	77.6	0.952
Female	78.3	71.8		77.3	77.3	
Age group (years)						
< 65	79.1	72.5	0.248	68.8	68.8	**0.039**
≥ 65	88.9	82.1		86.7	86.7	
Topography						
Colon	83.3	74.4	0.430	75.7	75.7	0.403
Upper rectal	86.7	86.7		86.7	86.7	
CEA						
Normal (<5.0)	90.0	84.0	**0.03**	83.9	83.9	**0.014**
Increased (>5.0)	66.8	58.5		59.4	59.4	
T stage						
T1-T2	95.7	95.7	**<0.001**	97.9	97.9	**<0.001**
T3-T4	72.1	40.1		56.7	56.7	
N stage						
N0	94.9	94.9	**<0.001**	91.2	91.2	**<0.001**
N+	66.6	35.5		55.8	55.8	
M stage						
M0	90.1	90.1	**<0.001**	86.1	86.1	**<0.001**
M+	49.0	0		28.6	28.6	
Histological grade						
Well differentiated	87.5	12.5	0.126	87.5	87.5	**0.026**
Moderately differentiated	53.3	0		78.8	78.2	
Poorly differentiated	0	0		40.0	40.0	
Blood embolization						
No	85.6	78.6	**0.002**	80.0	80.0	**<0.001**
Yes	33.3	0		0	0	
Lymphatic embolization						
No	85.1	60.3	0.151	80.3	80.3	0.079
Yes	76.9	0		58.3	58.3	
Perineural invasion						
No	84.6	84.6	**0.032**	79.8	79.8	0.108
Yes	0	0		55.6	55.6	
*KRAS*						
Wild	79.8	79.8	0.310	74.2	74.2	0.710
Mutated	61.9	61.9		66.7	66.7	
*Primer Pair 1- ERβ1, ERβ2* and ERβ4 isoforms*						
> −7.2	–	–		76.4	76.4	0.301
≤ −7.2	–	–		100	100	
> −3.2	87.5	87.5	0.361	–	–	
≤ −3.2	82.7	73.5		–	–	
*Primer Pair 2 - ERβ1* and ERβ5 isoforms*						
> −4.5	–	–		62.5	62.5	**0.012**
≤ −4.5	–	–		85.3	85.3	
> −7.6	81.6	81.6	0.118	–	–	
≤ −7.6	100	100		–	–	
Primer Pair 3 - ERβ 2 isoform*						
> −4.5	–	–		76.8	76.8	0.609
≤ −4.5	–	–		85.7	85.7	
> −3.2	82.4	75.2	0.187	–	–	
≤ −3.2	100	100		–	–	

*p*-value obtained by the log-rank test. Statistically significant *p* values (*p* < 0.05) are shown in bold type. *The cut-off for relative gene expression (log_2_) was determined using the log-rank test (maximally selected rank statistics in R) [24]

**Table 4:** Multivariate analysis of the prognostic factors of disease-free survival in colorectal cancer patients

Variable	N	HR	*p*	95% CI
T stage (TNM)				
T1-T2	47	1.0	**0.003**	2.88–170.7
T3-T4	51	22.1		
N stage (TNM)				
N0	59	1.0	**0.034**	1.09–8.41
N+	39	3.03		
*Primer Pair 2 – ERβ1* and *ERβ5 isoforms* (loss of relative gene expression (log_2_)^*^				
≤ −4.5	65	1.0	**0.002**	1.69–9.84
> −4.5	33	4.08		

*The cutoff for relative gene expression (log_2_) was determined using the log-rank test (maximally selected rank statistics in R) [24]

The cumulative probability of overall survival was 83.9% at 5 years and 77.1% at 10 years. The univariate analysis revealed greater overall survival in patients with normal serum carcinoembryonic antigen levels, T1-T2 tumours and the absence of perineural, lymph node or metastatic invasion. ERβ expression levels were not statistically significant in predicting overall survival (Table 3).

## Discussion

The effects of oestrogens on tissue are mediated by members of the nuclear oestrogen receptor subfamilies ERα and ERβ. In normal colorectal tissues, ERβ is predominant and appears to play an important role in the biological mechanisms of action of sex steroids [25, 26]; its expression decreases at higher Dukes’ stages, which display little or no ERα expression [9–11, 19]. Despite being important in the development of breast cancer, ER*α* is found in low levels in normal colorectal tissue [26]. Using different strategies, several studies reported no differences between the expression levels of ERα comparing colorectal tumours and normal mucosa [12, 13, 27–29]. Therefore, the ERβ variant isoforms may be the key to understand the ERβ signalling in different tissues [17].

The expression of splicing variants in colon cancer cells was first reported by Campbell-Thomson et al. in 2001 [19]. In normal human colon tissue, *ERβ1, ERβ2* and *ERβ5* are expressed, whereas *ERβ3* and *ERβ4* have been found only in testicles. In 2005, Wong et al. reported the expression of *ERβ* isoforms in colorectal carcinomas [30]. In that study, the expression levels of *ERβ1* and *ERβ2* were completely lost in 22% and 49% of primary colorectal carcinomas, respectively. In contrast, a truncated isoform referred to as *ERβ5* was found in all colorectal carcinomas and in tumour cell lines. Despite the limited number of cases, the authors suggested a prognostic role of *ERβ1* and *β2* isoforms.

A few alternative *ERβ1* isoforms (*ERβ2/ERβcx, ERβ3, ERβ4* and *ERβ5*) that result from alternative splicing of the last receptor-encoding exon have been reported. These proteins have a truncated domain or are otherwise altered in the C-terminal region [31]. The C-terminal region of the ER contains a ligand-binding domain that is involved in transcription activation, receptor dimerization, nuclear translocation and interaction of the receptor with transcription co-regulators [32]. Therefore, modifications in this region can affect the activity and biological function of the ER. Leung et al. (2006) reported that ERβ2–4-5 isoforms are deficient in intrinsic ligand-dependent transactivation activity, making ERβ1 (wild type) the only functional receptor isoform [17]. However, ERβ2–4-5 isoforms form heterodimers with ERβ1 and may increase ERβ1-induced transcription activity at physiological oestrogen concentrations.

In tissues such as prostate, breast and endometrium, a relationship between oestrogen signalling and the initiation of carcinogenesis, cancer progression and therapeutic response have been suggested [16, 33–38]. Oestrogenic action and the quantitative variation in the proportion of ERβ in different tissues indicate that the ERβ subtypes have different functions [35, 39]. Although the specific roles of ERβ subtypes in colorectal cancer are still uncertain, it is plausible that the varying co-expression of different isoform groups in normal mucosa, polyps and tumours of the colon, as shown in this study, can alter carcinogenesis. These findings should be taken into account in the design of future studies and screening protocols. In addition, the frequency of screening examinations should be re-evaluated in subjects who are undergoing hormone replacement or hormone deprivation therapy, treatments that are commonly used in breast cancer. These patients have a higher risk of developing colorectal cancer, and the hormonal exposure they receive may be involved in this increased risk. The effect of the use of hormone replacement therapy in patients with CRC or FAP syndrome must therefore be carefully evaluated.

The analysis to evaluate *ERβ* isoforms levels was performed in fresh frozen tissues (sporadic tumours) and in FFPE samples (sporadic and FAP polyps). One limitation of our study is the use of FFPE samples, which presents RNA with lower quality compared to fresh frozen tissue samples. However, the comparison of the expression levels of different *ERβ* isoforms using normal mucosa (fresh frozen tissue) and normal FAP mucosa (FFPE) revealed decreased *ERβ* expression in tumour samples and polyps. This result was confirmed grouping all samples independently of the tissue type (fresh tissue or FFPE). To validate the data, RNA-Seq data from 285 colon adenocarcinomas and 41 non-tumour colon tissue samples extracted from the TCGA dataset were used. In agreement with our data, this analysis revealed that mRNA ERβ expression levels were significantly lower in tumour tissue than in non-tumorous tissue samples (*p* < 0.001; Mean Diff 0.984 ± 0.146; 95% CI 0.697 to 1.271). Nguyen-Vu et al. (2016) using RNA-seq data of 233 colon adenocarcinomas and 21 non-tumor colon tissues from The Cancer Genome Atlas (TCGA) dataset. The ERβ expression was decreased in the cancerous state compared to non-cancerous tissues [40].

In our study, *ERβ1, ERβ2* and *ERβ4* variants (primer sets 1 and 3) showed downexpression in ST compared to NS. Similarly, *ERβ1, ERβ2, ERβ4* and *ERβ5* variants (primer sets 1, 2 and 3) presented downexpression in FAP polyps compared with NFAP. These findings were confirmed with TCGA dataset results in *ERβ2* and *ERβ4* performed in sporadic tumours. However, these transcripts demonstrated very low expression by RNA sequencing and some variants was not even detected by TCGA (i.e.: *ERβ1* and *ERβ5*). Although RNA sequencing is a suitable method to identify and discriminate mRNA variants, the sensitivity of RT-qPCR is superior, which is considered a gold standard methodology to evaluate transcripts [41–43].

Primer pair 2, which amplifies the transcript variants a and g (encoding *ERβ*1 and *ERβ5*) showed the highest expression levels of *ESR2* in normal FAP mucosa (NFAP). FAP polyps emerge during puberty and increase in number during adolescence, which is the period of peak oestrogen production. Based on these findings, it appears that the transcript variants have the potential to act as cofactors in the development of polyps and FAP-related colorectal carcinogenesis. Interesting, comparing sporadic tumours (ST) with sporadic normal mucosa (NS), the *ERβ*1 and *ERβ5* levels were not significant.

A primary chemoprevention trial [44] consisting of a double-blinded, randomised four-year study using sulindac included subjects with *APC* mutations as well as phenotypically unaffected subjects. The authors reported total eradication of the polyps in the placebo group, which included one patient who received an occasional administration of oral contraceptives and who had developed polyps [44]. The patient follow-up comprised flexible rectosigmoidoscopy every four months for 48 months, and an increased prevalence and recurrence of polyps was observed after the discontinuation of oral contraceptives.

Selective oestrogen receptor modulators (SORMs or SERMs) such as raloxifene and tamoxifen have both oestrogenic and anti-oestrogenic actions depending on the tissue type [44–46]. For example, raloxifene is not only effective in preventing osteoporosis [47] but has also been shown to be as effective as the SORM archetype tamoxifen in preventing breast cancer [48]; however, it increases the risk of uterine adenocarcinoma [49]. Despite the widespread clinical application of these modulators, very little is known about how they may affect colon cancer. It is important to identify oestrogen receptor variant subtypes and to determine their relative expression levels at different stages of tumorigenesis, especially in different tissues, because specific oestrogen receptor subtypes can be involved in the specific agonistic or antagonistic actions of oestrogens. The next step is therefore to determine the correlation between the predominance of specific isoforms and their interactions with various forms of oestrogen (isoflavones, SORM and hormone replacement therapy). The incidence of colorectal cancer is much lower in Asian countries than in the western world, a fact that may be explained by the high intake of phyto-oestrogens, such as soy, in Asian cultures [50]. This intake offers a protective effect that may be associated with the mediation of ER binding to genistein, which is the main phyto-oestrogen in soy and is thought to be a tyrosine kinase inhibitor, a characteristic that may explain some of its action on the colon [51, 52].

When invasive tumours were analysed individually, no differences in expression associated with the degree of tumour differentiation were found. This result likely occurred because the majority (87.8%) of the tumours studied herein displayed a moderate degree of differentiation. Immunohistochemistry analysis using an ERβ monoclonal antibody revealed decreased expression in higher grade or more dedifferentiated tumours [11, 13].

Although the results of a comparison of individual T stages (T1 vs T2 vs T3 vs T4) were not significant, most likely due to the limited number of cases at stages T1 and T4, the grouped analysis revealed that larger (T3/T4) tumours presented decreased ER*β* expression levels compared to T1/T2 tumours. These alterations were more evident and significant for the primer pair 1, which amplified the largest number of transcription variants (a, b, d, f, k and l), representing *ERβ1, ERβ2* and *ERβ4* isoforms. The amplification with the primers 2 and 3 resulted in a significant reduction of the expression levels in normal mucosa to very low values during the colorectal carcinogenesis, which may explain the absence of differences between T1/T2 and T3/T4 tumours. One plausible explanation is that the primer 1 is more sensitive for the detection of these differences, because contains a group with more homologous variants. The loss of ERβ expression was significantly different in adenomatous polyps with low-grade dysplasia, illustrating that differences in the expression of these receptors occur at early stages of carcinogenesis.

Using an expression level threshold of −4.5, we found that low expression levels of the *ERβ1* and *ERβ5* isoforms (transcript variants a and g: primer pair 2) were independent factors associated with sporadic colorectal cancer recurrence (ERβ > −4.5 presented a 4.08-fold higher risk of relapse (CI 1.69–9.84); *p* = 0.002**)**. Paradoxically, this sample set of cases displayed no significant difference in loss of normal mucosa ERβ expression. With respect to polyps and smaller and larger sporadic tumours, this subgroup was different in tissues from patients with FAP syndrome; therefore, it is plausible that this isoform group may be correlated with the APC suppressor gene, which is mutated in FAP syndrome. In contrast, sporadic cases are often associated with other variables associated with somatic carcinogenesis.

Cases of FAP cancer were not included in this study, which was designed to evaluate the prognosis of FAP tumours, because FAP is a very rare syndrome and therefore involves a heterogeneous group of patients; as a result, cases of FAP have been historically treated in several ways, which may lead to uncertainties in the prognostic results. Unfortunately, the gold standard for preventive treatment of colorectal cancer in FAP individuals is still total prophylactic colectomy at a young age, preferably prior to the malignant transformation of polyps [7]. Thus, we investigated mRNA expression of ERβ isoforms in normal mucosa and polyps. Significant loss was demonstrated in all subgroups of ERβ isoforms in adenomatous polyps of PAF compared to normal mucosa (*p* < 0.001).

Recently, an alternative mechanism for oestrogenic action was described where the G protein-coupled oestrogen receptor (GPER) mediates the rapid non-genomic effects of oestrogen, phyto-oestrogen and xeno-oestrogen [53]. GPER activation may inhibit the growth of CRC cells, both in vitro and in vivo, through multiple intracellular signalling pathways. Liu et al. (2017) showed that GPER expression in tumour tissue was markedly lower than in corresponding adjacent normal mucosal tissue. Tumours expressing lower levels of GPER exhibited a significantly lower survival rate than those with higher GPER expression levels [54]. The physiological significance of these rapid effects and their integration with nuclear responses to oestrogen are important issues that still need to be further investigated.

## Conclusions

Overall, we demonstrated that the mRNA expression levels of ERβ isoforms are downregulated in sporadic colorectal cancer and in FAP individuals. T3/T4 tumours also presented decreased expression of ERβ. Additionally, the expression levels of *ERβ1* and *ERβ5* were associated with the probability of disease-free survival. These differences may inform new clinical studies aimed at preventative strategies, especially in groups of patients who are receiving hormone therapy or are under conditions of hormonal deprivation. Although the limitation of our study was the analysis of the gene expression levels in a set of FFEP specimens, our data pointed out that the deregulation of *ESR2* isoform variants may be associated with colorectal cancer progression.

## Additional files

Additional file 1: Table S1. Geometric mean (GM) of the Cqs values of the reference transcripts and the quantification/quality information of the RNA samples. Samples with a mean Cq greater than the mean + 1 standard deviation of the GM of the Cqs of the three reference genes were excluded (XLSX 40 kb)

Additional file 2: Figure S1. *ESR2* expression levels in normal mucosa and polyps of FAP patients and in sporadic colon carcinomas. All groups of samples were compared without consider the sample type (Fresh frozen tissue or FFPE). NS = Sporadic normal mucosa; SP = Sporadic polyps; ST = Sporadic tumour; NFAP = Normal mucosa FAP; PFAP = Polyp FAP; *: *p* < 0.05; **: *p* < 0.01; ***: *p* < 0.001; (Tukey’s Multiple Comparison Test and Student’s t-test) (PNG 86 kb)

Additional file 3: Figure S2. *ESR2* isoforms expression levels in normal and in adenocarcinoma colon tissues deposited in the TCGA database. The isoforms most prevalent identified as uc001xgy.2 (green), uc001xgu.3 (blue), uc001xgx.3 (light blue), uc001xgw.3 (pink), and uc001xgz.2 (purple) coding (pink), and No-coding (purple) represents the transcripts variants and isoforms d (ERβ4), b (ERβ2), j (Non-coding RNA – (NC)), h (NC) and e (NC), respectively (PNG 106 kb)

Additional file 4: Figure S3. Kaplan − Meier estimate and cumulative incidence curve of the disease-free survival of colorectal cancer patients as a function of ERβ1 and ERβ5 isoform expression levels (primer pair 2: transcript variants a and g). The cutoff for relative gene expression of −4.5 (log2) was determined using the log-rank test (maximally selected rank statistics in R) [24] (PNG 86 kb)

## Abbreviations

Cq: Quantification Cycle
CRC: Colorectal Cancer
ERβ: Oestrogen Receptor Beta
ESR2: Oestrogen Receptor Beta Gene
FAP: Familial Adenomatous Polyposis
NFAP: Normal Mucosa from FAP Patients
NS: Sporadic Normal Mucosa
PFAP: Adenomatous Polyps from FAP Patients
SD: Standard Deviation
SP: Sporadic Polyps
ST: Sporadic Tumours
TCGA: The Cancer Genome Atlas
TNM: Tumour-Node-Metastasis Staging System
