# Supplementary figures and images for: Oestrogen receptor beta isoform expression in sporadic colorectal cancer, familial adenomatous polyposis and progressive stages of colorectal cancer
Source: BMC Cancer. 2017 Nov 13;17:754. doi: 10.1186/s12885-017-3688-4 (PMC5683223; doi:10.1186/s12885-017-3688-4)

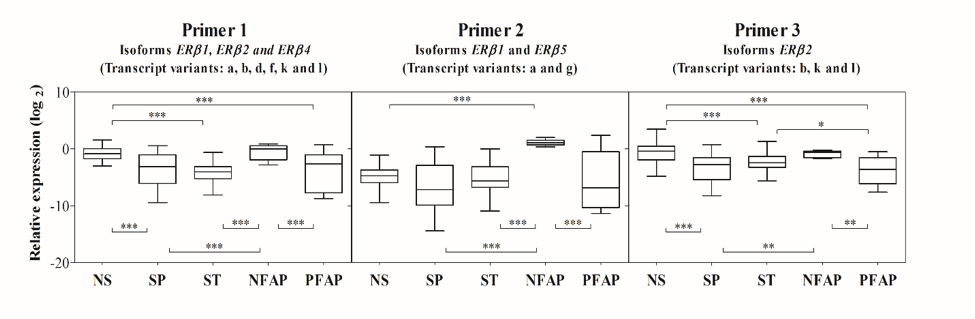

Supplement: Supplementary file 2 — ESR2 expression levels in normal mucosa and polyps of FAP patients and in sporadic colon carcinomas. All groups of samples were compared without consider the sample type (Fresh frozen tissue or FFPE). NS = Sporadic normal mucosa; SP = Sporadic polyps; ST = Sporadic tumour; NFAP = Normal mucosa FAP; PFAP = Polyp FAP; *: p < 0.05; **: p < 0.01; ***: p < 0.001; (Tukey’s Multiple Comparison Test and Student’s t-test) (PNG 86 kb) [file 12885_2017_3688_MOESM2_ESM.png]

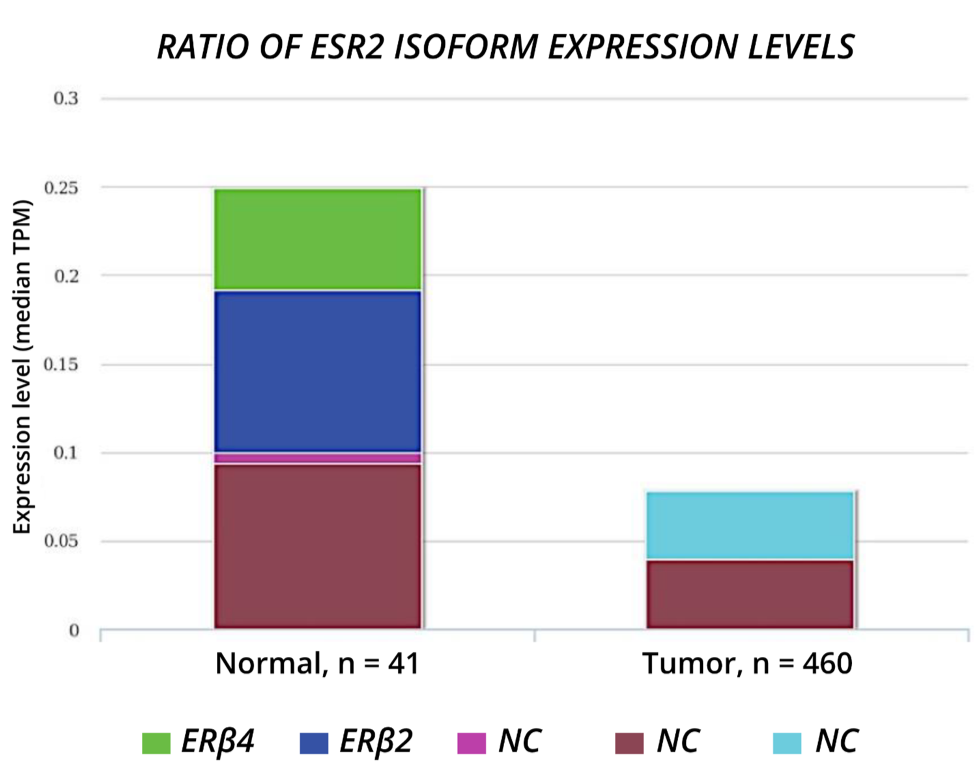

Supplement: Supplementary file 3 — ESR2 isoforms expression levels in normal and in adenocarcinoma colon tissues deposited in the TCGA database. The isoforms most prevalent identified as uc001xgy.2 (green), uc001xgu.3 (blue), uc001xgx.3 (light blue), uc001xgw.3 (pink), and uc001xgz.2 (purple) coding (pink), and No-coding (purple) represents the transcripts variants and isoforms d (ERβ4), b (ERβ2), j (Non-coding RNA – (NC)), h (NC) and e (NC), respectively (PNG 106 kb) [file 12885_2017_3688_MOESM3_ESM.png]

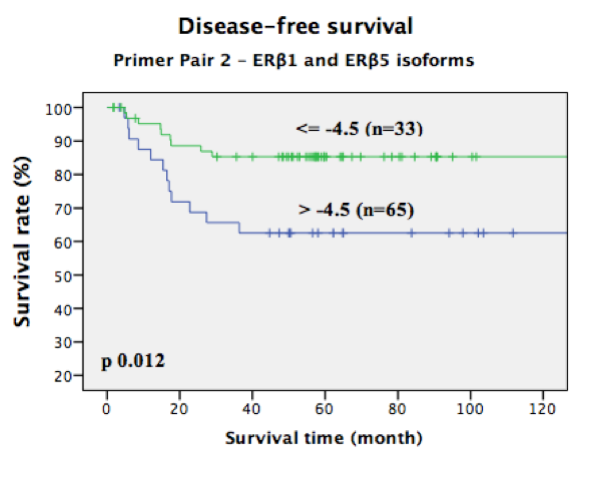

Supplement: Supplementary file 4 — Kaplan − Meier estimate and cumulative incidence curve of the disease-free survival of colorectal cancer patients as a function of ERβ1 and ERβ5 isoform expression levels (primer pair 2: transcript variants a and g). The cutoff for relative gene expression of −4.5 (log2) was determined using the log-rank test (maximally selected rank statistics in R) [24] (PNG 86 kb) [file 12885_2017_3688_MOESM4_ESM.png]
